# The challenges of preventing bovine tuberculosis

**DOI:** 10.2471/BLT.18.020218

**Published:** 2018-02-01

## Abstract

Bovine tuberculosis destroys livelihoods and is hampering efforts to achieve the End TB Strategy goal of tuberculosis elimination by 2030. Sophie Cousins reports on efforts to stamp out this neglected infectious disease.

It’s early morning in the Mexican state of Jalisco, and Zapopan’s municipal slaughterhouse is already in full swing.

Dozens of cows are lined up. Within just three hours, 200 of them have been slaughtered. Two veterinarians wearing orange helmets are tasked with inspecting the cows for bovine tuberculosis.

Every cow trucked in from ranches across the state is checked after death for lesions on their lymph nodes in their head, respiratory or digestive tract and other organs.

If a cow has suspected tuberculosis lesions, a sample is taken to a nearby laboratory to confirm whether the lesion is due to bovine tuberculosis or not.

It’s an onerous process, but one that is vital to ensure that infected animal products are not passed through the food supply to humans.

“Looking for and finding tuberculous lesions – as part of slaughter house inspection – helps to guarantee that the bovine products are safe for human consumption,” says Dr Alejandro Perera Ortiz, an agricultural specialist from the United States Department of Agriculture, based in Mexico City.

“This basic surveillance is important, as it helps us to locate infected herds and to take measures to prevent further spread of the disease among ranches,” he says, referring to the slaughter of infected herds to contain the infection. 

What happens in this Mexican slaughterhouse illustrates one way to prevent the spread of bovine tuberculosis from animals to humans.

Between 2015 and 2016, 179 countries and territories reported their status with regards to bovine tuberculosis to the World Organisation for Animal Health (OIE). More than half of these locations reported the disease in livestock and/or wildlife, demonstrating its wide geographical spread.

Unlike the main form of tuberculosis, which is caused by the bacterium, *Mycobacterium tuberculosis*, and is spread from person to person, zoonotic tuberculosis is caused by *Mycobacterium bovis* and is spread from animal to human. This occurs mainly through the consumption of unpasteurized dairy products, but also – less commonly – through the consumption of raw or uncooked meat or direct physical contact with infected animals.

“Heat-treatment of milk is key to reducing the risk to people,” says Dr Anna Dean from the World Health Organization’s (WHO) Global Tuberculosis Programme in Geneva.

“Targeting bovine tuberculosis in this way also brings benefits for the prevention of other foodborne diseases, such as those caused by *Brucella, Campylobacter*,* Escherichia coli, Salmonella *and *Listeria *species,” Dean says.

“Targeting bovine tuberculosis in this way also brings benefits for the prevention of other foodborne diseases.”

Anna Dean

There were an estimated 147 000 new cases of zoonotic tuberculosis and some 12 500 people died of the disease in 2016. Africa carries the largest burden of human cases, followed by Asia.

In 2016, there were almost 1.7 million deaths from tuberculosis and 10.4 million new infections overall, according to the WHO’s 2017 *Global tuberculosis report*.

While zoonotic tuberculosis cases make up only a small proportion of the overall human tuberculosis disease burden, ending the tuberculosis epidemic by 2030 – the goal of WHO’s End TB Strategy – will not be possible without combating zoonotic tuberculosis.

“In order to achieve the targets of the strategy, we must find and treat every patient with tuberculosis,” Dean explains.

“However, finding every case of zoonotic tuberculosis raises many challenges. For one, health-care workers have few diagnostic tools to differentiate *M. bovis* from *M. tuberculosis *in humans, which means that the true burden of the disease in humans is unknown and may be underestimated,” she says.

Another major challenge is that zoonotic tuberculosis is resistant to one of the first-line anti-tuberculosis drugs, making treatment of humans with zoonotic tuberculosis difficult in an age of increasing drug resistance.

The One Health approach which integrates efforts to improve health at the interface between people, animals and the environment is key to tackling zoonotic tuberculosis.

In recognition of this, WHO, along with other international agencies including the OIE, the Food and Agriculture Organization of the United Nations (FAO) and the International Union Against Tuberculosis and Lung Disease, launched a multidisciplinary *Roadmap for zoonotic tuberculosis* in November last year.

The Roadmap sets goals for 2020 and 2025 in three areas: improving the scientific base, reducing transmission at the animal–human interface and strengthening inter-sectoral and collaborative approaches.

Some of the 2020 goals include improved capacity of national health-care and laboratory services for diagnosing and treating zoonotic tuberculosis; scaled-up efforts to improve national food safety standards; and for zoonotic and bovine tuberculosis to be properly addressed by government authorities.

The 2025 targets are more ambitious and include new, rapid diagnostic tools for zoonotic tuberculosis to be rolled out to high-risk groups, the development of anti-tuberculosis vaccines for humans and an effective bovine tuberculosis vaccine for livestock.

“An effective bovine tuberculosis eradication programme is needed to reduce and eventually eliminate its transmission to human populations,” says Perera Ortiz, who is also Chair of the zoonotic tuberculosis sub-section at the International Union Against Tuberculosis and Lung Disease.

For Dr Mario Raviglione, who was the director of the WHO’s Global Tuberculosis Programme until November last year, the Roadmap brings zoonotic tuberculosis, a previously neglected disease, into the spotlight, which is positive. He points out, however, that success hinges on political commitment, which he describes as “difficult”.

“Whether the goals of the Roadmap can be achieved will depend on political commitments, engagements with heads of state and financing,” he says.

Dr Matthew Stone, Deputy Director-General of the OIE, agrees. “It’s clear that these are aspirational targets with significant challenges,” he says, referring to the Roadmap.

Stone stresses that more should be done to improve food safety standards and to control bovine tuberculosis in the animal reservoir.

Many countries where the disease is endemic lack qualified veterinarians and systems to identify and control bovine tuberculosis in animals and good milk pasteurization systems. Wildlife can also play an important role in maintaining the disease in livestock.

Moreover, many rural communities lack knowledge about the disease and often live in close proximity to animals: both factors that fuel the spread of disease.

“The biggest challenge is building the regulatory capacity of veterinary services and successfully promoting awareness and behavioural change in livestock owners,” Stone says.

“Veterinary services may have critical weaknesses in many countries where the disease is endemic; for instance, animal health surveillance to detect and control infected herds may be insufficient, meat inspections may not be carried out by trained professionals and implementation of important food safety policies such as pasteurization may be weak.”

In 2011, Timpiyian Leseni from Kajiado, 80 kilometres south of Kenya’s capital, Nairobi, where she is a member of the Maasai community, noticed her stomach was increasing in size every day. Then the night sweats, vomiting, nausea and weight loss began.

Leseni was admitted to hospital in Nairobi and underwent an operation to remove pus from her intestines. When the doctor told her the cause of the masses in her stomach was tuberculosis, she was shocked.

“’What do you mean tuberculosis of the intestines?’ I asked the doctor. He told me that this type of tuberculosis was from drinking milk that had not been boiled or from eating meat that wasn’t cooked properly,” she says.

“We drink blood straight from the goat [after its slaughter] for nutritional reasons. We were brought up doing this,” she says. “But not anymore. I’ve stopped.”

After recovering from tuberculosis, Leseni was inspired to educate her community about the importance of boiling milk and ensuring that meat products are cooked well.

“How can I tell them that what they have been always eating may be infected?”

Timpiyian Leseni

She also wanted to dispel myths about how human tuberculosis is spread. But, changing deep-seated human behaviour is also a major challenge. “How can I tell them that what they have been always eating may be infected?” she says.

Over time, however, word spread in the community and more people came forward to tell their stories of how they’d lost loved ones due to the same symptoms that Leseni had experienced.

Leseni now runs a community-based organization called Talaku Community Based Organization, which collaborated with WHO in 2012 on the Engage TB pilot project to involve communities in the tuberculosis response.

For Dr Berhe Tekola, Director of the FAO Animal Production and Health Division, it is vital for governments to recognise the interdependence between human and animal health in the fight against tuberculosis. 

“Bovine tuberculosis threatens people’s livelihoods and results in major economic and trade barriers, as well as posing a risk to food safety and human health,” Tekola says. 

**Figure:**
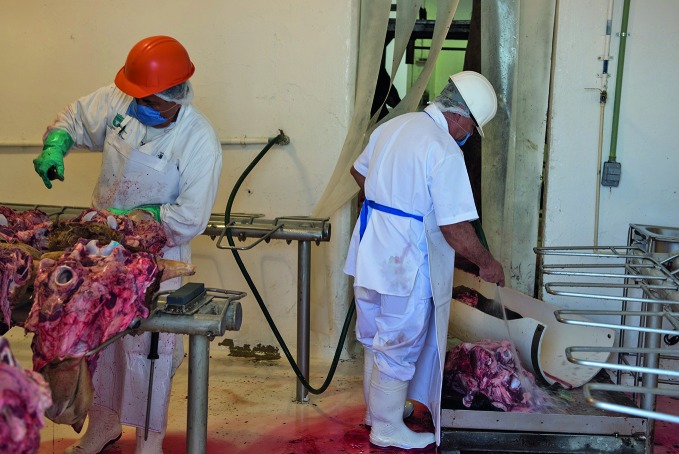
Veterinarian wearing an orange helmet inspects a slaughtered animal for signs of bovine tuberculosis in Zapopan, Mexico.

Steve Forrest

**Figure:**
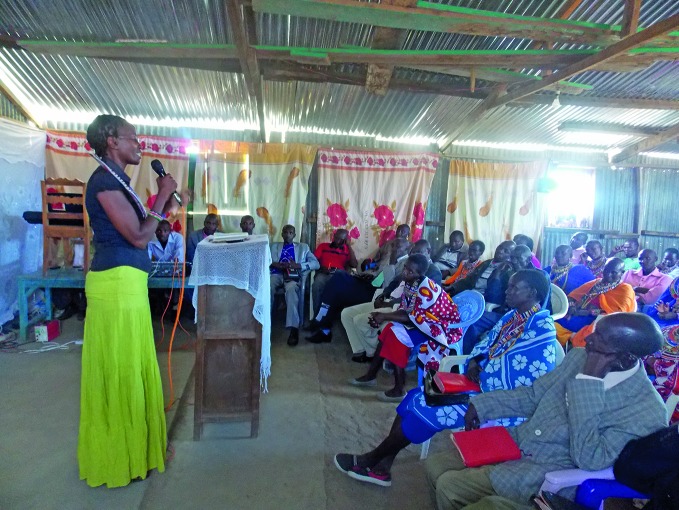
Timpiyian Leseni talks to fellow members of the Maasai tribe in the United Republic of Tanzania to raise awareness about bovine tuberculosis.

Courtesy of Timpiyian Leseni

